# Crystal structure of betaine aldehyde dehydrogenase from *Burkholderia pseudomallei*

**DOI:** 10.1107/S2053230X21013455

**Published:** 2022-01-27

**Authors:** Dylan K. Beard, Sandhya Subramanian, Jan Abendroth, David M. Dranow, Thomas E. Edwards, Peter J. Myler, Oluwatoyin A. Asojo

**Affiliations:** a Department of Chemistry and Biochemistry, Hampton University, 100 William R. Harvey Way, Hampton, VA 23668, USA; b Center for Global Infectious Disease Research, Seattle Children’s Research Institute, 307 Westlake Avenue North Suite 500, Seattle, WA 98109, USA; c Seattle Structural Genomics Center for Infectious Disease (SSGCID), Seattle, Washington, USA; d UCB-Bainbridge, Bainbridge Island, WA 98110, USA; e Department of Global Health and Department of Biomedical Informatics and Medical Education, University of Washington, Seattle, WA 98195, USA

**Keywords:** *Burkholderia pseudomallei*, melioidosis, disulfiram, betaine aldehyde dehydrogenase, inhibition, drug repurposing

## Abstract

*Burkholderia pseudomallei* infection causes melioidosis, which is often fatal if untreated. There is a need to develop new and more effective treatments for melioidosis. This study reports apo and cofactor-bound crystal structures of the potential drug target betaine aldehyde dehydrogenase (BADH) from *B. pseudomallei*. A structural comparison identified similarities to BADH from *Pseudomonas aeruginosa* which is inhibited by the drug disulfiram. This preliminary analysis could facilitate drug-repurposing studies for *B. pseudomallei*.

## Abstract 2

High-resolution apo and cofactor-bound structures of betaine aldehyde dehydrogenase from *Burkholderia pseudomallei* are described.

## 1. Introduction

*Burkholderia pseudomallei* is a rod-shaped, motile, flagellated, soil-dwelling Gram-negative proteobacterium of the Burkholderiaceae family that thrives in tropical and sub­tropical regions (Gassiep *et al.*, 2021 ▸). *B. pseudomallei* causes melioidosis, a deadly emerging opportunistic infection mainly of the immunocompromised (Hall *et al.*, 2019 ▸; Poe *et al.*, 1971 ▸; Veluthat *et al.*, 2021 ▸). *B. pseudomallei* is transmitted through open wounds, contact with contaminated soil and water, ingestion or inhalation, and it is also a potential biological warfare agent (Goarant *et al.*, 2021 ▸). Melioidosis is endemic in South Asia and Northern Australia, with ∼165 000 cases annually; however, the global distribution of *B. pseudomallei* is unknown, as the associated disease is underreported and misdiagnosed (Patil *et al.*, 2016 ▸; Poe *et al.*, 1971 ▸; Veluthat *et al.*, 2021 ▸). Typically, about 12 cases of melioidosis are reported annually in mainland USA, and most patients had traveled internationally; however, *B. pseudomallei* occurs naturally in Puerto Rico and the US Virgin Islands (Hall *et al.*, 2019 ▸). Melioidosis symptoms include localized pain and swelling, ulcer, cough, headache, anorexia, joint pain, brain infection, seizures, fever, pneumonia, low blood pressure and abscesses (Hall *et al.*, 2019 ▸; Poe *et al.*, 1971 ▸; Veluthat *et al.*, 2021 ▸). Thus, melioidosis may be misdiagnosed as tuberculosis, pneumonia or other diseases (Veluthat *et al.*, 2021 ▸). Symptoms may appear a few days or several years after exposure, and the mortality rate of untreated melioidosis is around 90% (Loveleena *et al.*, 2004 ▸; Patil *et al.*, 2016 ▸; Poe *et al.*, 1971 ▸; Veluthat *et al.*, 2021 ▸). Melioidosis is currently treated with two to eight weeks of intravenous antimicrobial therapy (ceftazidime or meropenem) followed by 3–6 months of oral antimicrobial therapy (amoxicillin/clavulanic acid or trimethoprim–sulfamethoxazole), but still results in ∼40% mortality (Fen *et al.*, 2021 ▸). As a part of efforts to develop new therapeutics and diagnostics for melioidosis, the Seattle Structural Genomics Center for Infectious Disease (SSGCID) has determined the crystal structure of betaine aldehyde dehydrogenase (BADH) from *B. pseudomallei* (*Bp*BADH). BADH catalyzes the irreversible oxidation of betaine aldehyde to the osmoprotectant betaine and is being investigated as a drug target for drug-resistant bacteria, notably *Pseudomonas aeruginosa*, because its inhibition blocks choline catabolism and leads to the accumulation of the highly toxic betaine aldehyde (González-Segura *et al.*, 2009 ▸).

## 2. Materials and methods

### 2.1. Production of *Bp*BADH

Cloning, expression and purification were conducted as part of the Seattle Structural Genomics Center for Infectious Disease (SSGCID; Myler *et al.*, 2009 ▸; Stacy *et al.*, 2011 ▸) following standard protocols as described previously (Bryan *et al.*, 2011 ▸; Choi *et al.*, 2011 ▸; Serbzhinskiy *et al.*, 2015 ▸). The full-length betaine aldehyde dehydrogenase gene from *B. pseudomallei* (*Bp*BADH; UniProt Q3JLL8) encoding amino acids 1–489 was PCR-amplified from genomic DNA using the primers shown in Table 1 ▸.

The gene was cloned into the ligation-independent cloning (LIC; Aslanidis & de Jong, 1990 ▸) expression vector pMCSG26 (Eschenfeldt *et al.*, 2010 ▸) encoding a noncleavable C-terminal 6×His fusion tag (ORF-GHHHHHH). Plasmid DNA was transformed into chemically competent *Escherichia coli* BL21(DE3)R3 Rosetta cells. The plasmid containing Q3JLL8 was expression-tested, and 2 l of culture was grown using auto-induction medium (Studier, 2005 ▸) in a LEX Bioreactor (Epiphyte Three Inc.) as described previously (Serbzhinskiy *et al.*, 2015 ▸). The expression clone BupsA.00020.b.AE1.GE43326 is available at https://www.ssgcid.org/available-materials/expression-clones/.

**Figure 1:**
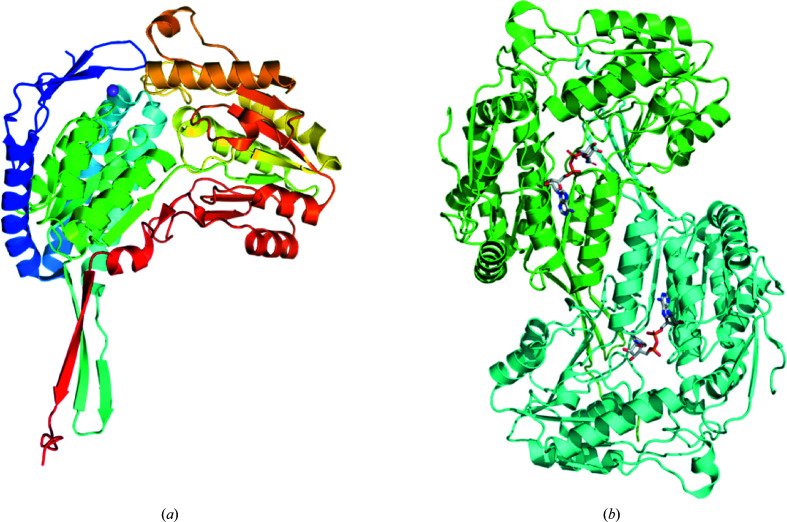
Structure of *B. pseudomallei* betaine aldehyde dehydrogenase (*Bp*BADH). (*a*) Monomer of apo *Bp*BADH (rainbow colored from blue at the N-terminus to red at the C-terminus. (*b*) Dimer of *Bp*BADH with NAD (monomers are shown as aquamarine and cyan ribbons, with NAD shown as sticks).

*Bp*BADH-His was purified using a two-step protocol consisting of an immobilized metal-affinity chromatography (IMAC) step and size-exclusion chromatography (SEC). All chromatography runs were performed on an ÄKTApurifier 10 (GE) using automated IMAC and SEC programs according to previously described procedures (Bryan *et al.*, 2011 ▸). Thawed bacterial pellets were lysed by sonication in 200 ml lysis buffer {25 m*M* 4-(2-hydroxyethyl)-1-piperazineethane­sulfonic acid (HEPES) pH 7.0, 500 m*M* NaCl, 5% glycerol, 0.5% 3-[(3-cholamidopropyl)dimethylammonio]-1-propanesulfonate (CHAPS), 30 m*M* imidazole, 10 m*M* MgCl_2_, 1 m*M* tris(2-carboxyethyl)phosphine hydrochloride (TCEP), 250 µg ml^−1^ 4-(2-aminoethyl)benzenesulfonyl fluoride hydrochloride (AEBSF), 0.025% sodium azide}. After sonication, the crude lysate was clarified with 20 µl (25 units µl^−1^) Benzonase and incubated while mixing at room temperature for 45 min. The lysate was clarified by centrifugation at 10 000 rev min^−1^ for 1 h using a Sorvall centrifuge (Thermo Scientific). For the IMAC step, the clarified supernatant was passed over an Ni–NTA HisTrap FF 5 ml column (GE Healthcare) which had been pre-equilibrated with loading buffer (25 m*M* HEPES pH 7.0, 500 m*M* NaCl, 5% glycerol, 30 m*M* imidazole, 1 m*M* TCEP, 0.025% sodium azide). The column was washed with 20 column volumes (CV) of loading buffer and was eluted with loading buffer and 250 m*M* imidazole in a linear gradient over 7 CV. Peak fractions, as determined by UV absorbance at 280 nm, were pooled and concentrated using an Amicon concentrator to a volume of 5 ml for SEC. For SEC, a SEC column (Superdex 75, GE) was equilibrated with running buffer [25 m*M* HEPES pH 7.0, 500 m*M* NaCl, 5% glycerol, 2 m*M* dithiothreitol (DTT), 0.025% sodium azide]. The eluted peak fractions were collected and analyzed for the presence of *Bp*BADH by SDS–PAGE. The SEC peak fractions containing *Bp*BADH eluted as a single large peak at a molecular mass of ∼77 kDa. A dimer of *Bp*BADH is expected to have a molecular mass of ∼106 kDa, while a monomer has a molecular mass of ∼53 kDa. *Bp*BADH may be a monomer in the absence of a cofactor, while it dimerizes in the presence of the cofactor or other ligands. Further biophysical analysis is required to determine whether *Bp*BADH dimerizes in the presence of NAD in solution. Interestingly, the dimer has been reported in more than 175 reported BADH structures with ligands, cofactors and inhibitors in the PDB and is consistent with the observed crystal form of *Bp*BADH with NAD (Fig. 1 ▸).

The peak fractions were pooled and concentrated to 34.72 mg ml^−1^ using an Amicon concentrator (Millipore). The protein concentration was assessed using the OD_280_ and a molar extinction coefficient of 46 870 *M* ^−1^ cm^−1^. Purified protein was aliquoted into 200 µl aliquots, flash-frozen in liquid nitrogen and stored at −80°C until use for crystallization. The purified protein (batch BupsA.00020.b.AE1.PS38619) is available at https://www.ssgcid.org/available-materials/ssgcid-proteins/.

### 2.2. Crystallization

Purified *Bp*BADH-His was screened for crystallization in 96-well sitting-drop plates against the JCSG++ HTS (Jena Bioscience), MCSG1 (Molecular Dimensions) and Morpheus (Rigaku Reagents; Gorrec, 2009 ▸, 2015 ▸) crystal screens. The protein solution for the apo structure did not contain NAD, whereas 4 m*M* NAD was added to the protein solution for the NAD-bound complex (Table 2 ▸). Equal volumes of protein solution (0.4 µl) and precipitant solution were set up at 287 K against reservoir (80 µl) in sitting-drop vapor-diffusion format. The final crystallization precipitant was JCSG+ condition F7 for the apo form and Morpheus condition H11 for the NAD-bound form (see Table 2 ▸). After cryoprotectant exchange into crystallization solution supplemented with 20% ethylene glycol, the crystals were harvested and flash-cooled by plunging them into liquid nitrogen.

### 2.3. Data collection and processing

Data were collected at 100 K on beamline 21-ID-F at the Advanced Photon Source (APS), Argonne National Laboratory (see Table 3 ▸). Data sets were reduced with *XSCALE* (Kabsch, 2010 ▸). Raw X-ray diffraction images are available from the Integrated Resource for Reproducibility in Macromolecular Crystallography at https://www.proteindiffraction.org/.

### 2.4. Structure solution and refinement

The structures were solved by molecular replacement with *Phaser* (McCoy *et al.*, 2007 ▸) from the *CCP*4 suite of programs (Collaborative Computational Project, Number 4, 1994 ▸; Krissinel *et al.*, 2004 ▸; Winn *et al.*, 2011 ▸) using PDB entry 2wox (Díaz-Sánchez *et al.*, 2011 ▸) as the search model. The structure was refined using iterative cycles of *Phenix* (Liebschner *et al.*, 2019 ▸) followed by manual rebuilding of the structure using *Coot* (Emsley & Cowtan, 2004 ▸; Emsley *et al.*, 2010 ▸). The quality of both structures was checked using *MolProbity* (Chen *et al.*, 2010 ▸). All data-reduction and refinement statistics are shown in Table 4 ▸. The structures of apo *Bp*BADH and *Bp*BADH with NAD were refined to resolutions of 2.05 and 1.55 Å, respectively. Structural figures were analyzed and prepared using *PyMOL* (DeLano, 2002 ▸). Multiple sequence alignments were generated with *Clustal Omega* (Li *et al.*, 2015 ▸). Coordinates and structure factors have been deposited in the Protein Data Bank (https://www.rcsb.org/; Berman *et al.*, 2000 ▸) with accession numbers 6wsa and 6wsb for apo *Bp*BADH and *Bp*BADH in complex with NAD, respectively.

## 3. Results and discussion

**Figure 2:**
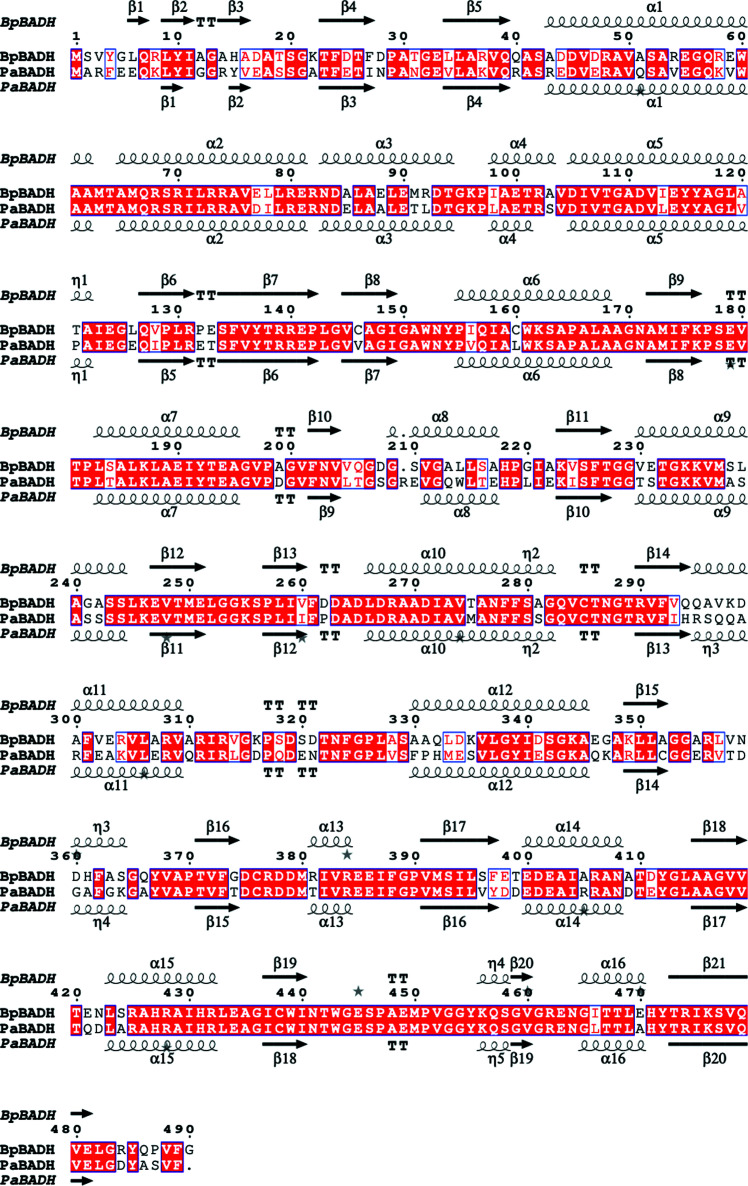
Structural and primary-sequence alignment of *Bp*BADH and *Pa*BADH. The secondary-structure elements shown are α-helices (α), 3_10_-helices (η), β-­strands (β) and β-turns (TT). Identical residues are shown in white on a red background and conserved residues are shown in red.

The two structures reported here are of apo *Bp*BADH and its complex with the cofactor NAD (Fig. 1 ▸). The monomers are very similar and have an r.m.s.d. of ∼0.17 Å for main-chain atoms. The 489 amino acids in each monomer fold as 20.4% β-strand, 39.3% α-helix, 2.5% 3_10_-helix and 37.8% loops, forming six β-α-β motifs that contain five β-sheets (four mixed and one antiparallel). The structure also contains 21 helices, 21 strands, four β-hairpins, four β-bulges and 25 helix–helix interactions. *Bp*BADH has a prototypical BADH topology and shares considerable structure and sequence similarity with the ortholog from *P. aeruginosa* (*Pa*BADH; Fig. 2 ▸). The 489-amino-acid sequence of *Pa*BADH folds as 19.6% β-strand, 38.2% α-helix, 2.5% 3_10_-helix and 39.7% loops (Fig. 2 ▸).

The structural similarities and motifs associated with the BADHs from both organisms may accelerate drug-discovery efforts. *Pa*BADH is known to be inhibited by disulfiram through the catalytic cysteine (Velasco-García *et al.*, 2006 ▸); thus, we hypothesize that *Bp*BADH will likewise be inhibited by disulfiram. Disulfiram binds irreversibly to Cys286 in *Pa*BADH, which is in the highly conserved cofactor-binding cavity of *Pa*BADH and *Bp*BADH; the corresponding residue is Cys285 in *Bp*BADH (Figs. 2 ▸ and 3 ▸). Disulfiram is an irreversible inhibitor that leads to a buildup of betaine aldehyde, which becomes toxic in bacterial cells. The toxicity in the bacterial cells stops bacterial growth (Velasco-García *et al.*, 2006 ▸). Since disulfiram is FDA-approved for treating chronic alcoholism, preliminary studies suggest that it could be repurposed as a lead compound for melioidosis. Furthermore, due to the structural similarity between *Pa*BADH and *Bp*BADH, the lessons learned in drug discovery for the former could accelerate efforts in addressing melioidosis.

## 4. Conclusion

The high-resolution structures of betaine aldehyde dehydro­genase from *B. pseudomallei* (*Bp*BADH) and *P. aeruginosa* (*Pa*BADH) reveal a conserved NAD-dependent topology and structural similarity. Since the key amino-acid residues in inhibitor-binding sites are conserved, the previous studies on *Pa*BADH could facilitate the development of small-molecule inhibitors of *Bp*BADH.

## Supplementary Material

PDB reference: betaine aldehyde dehydrogenase, 6wsa

PDB reference: bound to cofactor, 6wsb

## Figures and Tables

**Figure 3:**
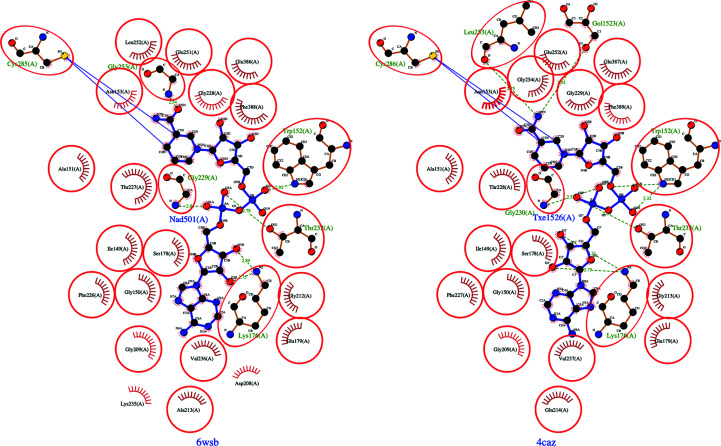
*LIGPLOT* analysis reveals that the cofactor-binding domains of *Bp*BADH (PDB entry 6wsb) and *Pa*BADH (PDB entry 4caz) are well conserved (circles indicate identical residues). Both structures show the conserved catalytic cysteine irreversibly inhibited by disulfiram.

**Table 1:** Macromolecule-production information

Source organism	*Burkholderia pseudomallei* 1710b
DNA source	Dr Samuel I. Miller, University of Washington, USA
Forward primer	5′-ATGTCCGTATACGGTCTGCAGC-3′
Reverse primer	5′-GAACACCGGTTGATAGCGGCC-3′
Expression vector	pMCSG26
Expression host	*E. coli* BL21(DE3)R3 Rosetta cells
Complete amino-acid sequence of the construct produced	MSVYGLQRLYIAGAHADATSGKTFDTFDPATGELLARVQQASADDVDRAVASAREGQREWAAMTAMQRSRILRRAVELLRERNDALAELEMRDTGKPIAETRAVDIVTGADVIEYYAGLATAIEGLQVPLRPESFVYTRREPLGVCAGIGAWNYPIQIACWKSAPALAAGNAMIFKPSEVTPLSALKLAEIYTEAGVPAGVFNVVQGDGSVGALLSAHPGIAKVSFTGGVETGKKVMSLAGASSLKEVTMELGGKSPLIVFDDADLDRAADIAVTANFFSAGQVCTNGTRVFVQQAVKDAFVERVLARVARIRVGKPSDSDTNFGPLASAAQLDKVLGYIDSGKAEGAKLLAGGARLVNDHFASGQYVAPTVFGDCRDDMRIVREEIFGPVMSILSFETEDEAIARANATDYGLAAGVVTENLSRAHRAIHRLEAGICWINTWGESPAEMPVGGYKQSGVGRENGITTLEHYTRIKSVQVELGRYQPVFGHHHHHH

**Table 2:** Crystallization

Method	Sitting-drop vapor diffusion
Plate type	96-well Compact 300, Rigaku
Temperature (K)	287
Protein concentration (mg ml^−1^)	34.72
Buffer composition of protein solution	
Apo crystals	25 m*M* HEPES pH 7.0, 500 m*M* NaCl, 5% glycerol, 2 m*M* DTT, 0.025% azide
NAD-bound crystals	25 m*M* HEPES pH 7.0, 500 m*M* NaCl, 5% glycerol, 2 m*M* DTT, 0.025% azide, 4 m*M* NAD
Composition of reservoir solution	
Apo structure	JCSG+ condition F7: 0.8 *M* succinate pH 7.0
NAD-bound structure	Morpheus condition H11: 10% PEG 4000, 20% glycerol, 0.02 *M* sodium L-glutamate, 0.02 *M* DL-alanine, 0.02 *M* glycine, 0.02 *M* DL-lysine, 0.02 *M* DL-serine, 0.1 *M* bicine/Trizma pH 8.5
Volume and ratio of drop	0.4 µl protein plus 0.4 µl reservoir
Volume of reservoir (µl)	80
Cryoprotectant	20% ethylene glycol

**Table 3:** Data collection and processing Values in parentheses are for the outer shell.

PDB code	6wsa	6wsb
Ligand	—	NAD
Diffraction source	21-ID-F, APS	21-ID-F, APS
Wavelength (Å)	0.97872	0.97872
Temperature (K)	100	100
Detector	RayoniX MX300HE CCD	RayoniX MX300HE CCD
Crystal-to-detector distance (mm)	270	200
Rotation range per image (°)	1	1
Total rotation range (°)	60	150
Space group	*P*6_2_22	*P*2_1_2_1_2
*a*, *b*, *c* (Å)	107.86, 107.86, 233.53	99.27, 156.70, 76.23
α, β, γ (°)	90, 90, 120	90, 90, 90
Mosaicity (°)	0.143	0.103
Resolution range (Å)	49.51–2.05 (2.10–2.05)	43.09–1.55 (1.59–1.55)
Total No. of reflections	362438 (26952)	1054995 (75766)
No. of unique reflections	51154 (3702)	172302 (12622)
Completeness (%)	99.9 (99.9)	99.9 (100.0)
Multiplicity	7.09 (7.28)	6.12 (6.00)
〈*I*/σ(*I*)〉	12.86 (3.50)	16.47 (3.02)
*R*_r.i.m._	0.101 (0.548)	0.083 (0.621)
Overall *B* factor from Wilson plot (Å^2^)	32.748	20.398

**Table 4:** Structure solution and refinement Values in parentheses are for the outer shell.

PDB code	6wsa	6wsb
Ligand	Glycerol	NAD
Resolution range (Å)	49.51–2.05 (2.08–2.05)	43.09–1.55 (1.57–1.55)
Completeness (%)	96.1	96.4
σ Cutoff	*F* > 0.000σ(*F*)	*F* > 0.000σ(*F*)
No. of reflections, working set	49171 (1957)	166209 (4765)
No. of reflections, test set	2932 (109)	10050 (297)
Final *R*_cryst_	0.140 (0.2203)	0.144 (0.2064)
Final *R*_free_	0.173 (0.2521)	0.169 (0.2342)
Cruickshank DPI	0.183	0.070
No. of non-H atoms		
Protein	3666	7322
Ion	1	—
Ligand	60	100
Solvent	501	1388
Total	4228	8810
R.m.s. deviations		
Bond lengths (Å)	0.006	0.006
Angles (°)	0.775	0.85
Average *B* factors (Å^2^)		
Protein	33.0	15.3
Ion	32.1	—
Ligand	58.9	31.7
Water	42.6	30.8
Ramachandran plot		
Most favored (%)	97.1	97.1
Allowed (%)	2.9	2.9
